# *Vital Signs*: Trends in *Staphylococcus aureus* Infections in Veterans Affairs Medical Centers — United States, 2005–2017

**DOI:** 10.15585/mmwr.mm6809e2

**Published:** 2019-03-08

**Authors:** Makoto Jones, John A. Jernigan, Martin E. Evans, Gary A. Roselle, Kelly M. Hatfield, Matthew H. Samore

**Affiliations:** ^1^Veterans Affairs Salt Lake City Healthcare System, Salt Lake City, Utah; ^2^Division of Epidemiology, University of Utah, Salt Lake City, Utah; ^3^Division of Healthcare Quality Promotion, National Center for Emerging and Zoonotic Infectious Diseases, CDC; ^4^MRSA/MDRO Prevention Office, National Infectious Diseases Service, Patient Care Services, Veterans Health Administration, Washington, D.C., ^5^National Infectious Diseases Service, Patient Care Services, Veterans Health Administration, Washington, D.C.

## Abstract

**Introduction:**

By 2007, all Department of Veterans Affairs medical centers (VAMCs) had initiated a multifaceted methicillin-resistant *Staphylococcus aureus* (MRSA) prevention program. MRSA and methicillin-susceptible *S. aureus* (MSSA) infection rates among VAMC inpatients from 2005 to 2017 were assessed.

**Methods:**

Clinical microbiology data from any patient admitted to an acute-care VAMC in the United States from 2005 through 2017 and trends in hospital-acquired MRSA colonization were examined.

**Results:**

*S. aureus* infections decreased by 43% overall during the study period (p<0.001), driven primarily by decreases in MRSA, which decreased by 55% (p<0.001), whereas MSSA decreased by 12% (p = 0.003). Hospital-onset MRSA and MSSA infections decreased by 66% (p<0.001) and 19% (p = 0.02), respectively. Community-onset MRSA infections decreased by 41% (p<0.001), whereas MSSA infections showed no significant decline. Acquisition of MRSA colonization decreased 78% during 2008–2017 (17% annually, p<0.001). MRSA infection rates declined more sharply among patients who had negative admission surveillance MRSA screening tests (annual 9.7% decline) compared with those among patients with positive admission MRSA screening tests (4.2%) (p<0.05).

**Conclusions and Implications for Public Health Practice:**

Significant reductions in *S. aureus* infection following the VAMC intervention were led primarily by decreases in MRSA. Moreover, MRSA infection declines were much larger among patients not carrying MRSA at the time of admission than among those who were. Taken together, these results suggest that decreased MRSA transmission played a substantial role in reducing overall *S. aureus* infections at VAMCs. Recent calls to withdraw infection control interventions designed to prevent MRSA transmission might be premature and inadvisable, at least until more is known about effective control of bacterial pathogen transmission in health care settings. Effective *S. aureus* prevention strategies require a multifaceted approach that includes adherence to current CDC recommendations for preventing not only device- and procedure-associated infections, but also transmission of health care–prevalent strains.

*On March 5, 2019, this report was posted as an *MMWR* Early Release on the *MMWR* website (*https://www.cdc.gov/mmwr*).*

## Introduction

*Staphylococcus aureus* is among the most common causes of health care–associated infections and accounts for significant morbidity and mortality. Beginning in 2005, in response to high rates of methicillin-resistant *S. aureus* (MRSA) infections, the U.S. Department of Veterans Affairs (VA) piloted an MRSA prevention program in 18 VA medical centers (VAMCs). By October 2007, all 153 VAMCs had implemented the MRSA prevention program, which included, among other components, admission screening for nasal MRSA carriage and using contact precautions (i.e., wearing a gown and gloves for all interactions involving contact with the patient or the patient's environment) for patients found to be carriers (*1*). To assess the impact of the intervention, the investigators tracked the incidence of MRSA and methicillin-susceptible *S. aureus* (MSSA) infections at 130 VAMCs from 2005 to 2017 and examined hospital-acquired MRSA colonization based on results of MRSA surveillance tests collected during the same period.

## Methods

Clinical data from any patient admitted to VAMCs in the United States from January 1, 2005 through December 31, 2017 were analyzed. Facilities were excluded from the study if they did not provide acute care or if they did not report data to VA’s periodic complexity assessment (e.g., the level and type of care provided) (*2*) in all eligible years during the study period. Clinical diagnostic culture and MRSA surveillance test results were extracted from electronic health records as described elsewhere (*3*). Bloodstream infections were defined as isolation of *S. aureus* from blood samples. Nonblood infections were defined as isolation of *S. aureus* from any other sample type, excluding those obtained for surveillance purposes and those obtained within 14 days of a positive blood culture. MRSA isolates from samples collected from the same patient within 365 days were considered duplicates and excluded; MSSA duplicates were defined in the same manner. Infections were classified as hospital-onset when the specimen was obtained >3 days after admission, and as community-onset when the specimen was obtained ≤3 days after admission. Community-onset infection rates and total (combined community-onset and hospital-onset) infection rates were calculated per admission. Hospital-onset infection rates were expressed per 1,000 patient-days-at-risk, excluding days after the patient had met one of the infection definitions. MRSA colonization status at admission was considered positive if the last test within 24 hours after admission was positive. Patients were considered to have acquired MRSA if they had at least one MRSA-positive test (clinical or surveillance) after a negative admission surveillance test. Fluoroquinolone use was measured and defined according to National Healthcare Safety Network methods to assess for potential changes in antimicrobial pressure exerted on *S. aureus* (*4*).

To model rates, trend analyses were performed with generalized estimating equation models clustering by VAMC and using a negative binomial distribution, patient days at risk as the exposure, an autoregressive correlation structure, and robust error estimation. Models were adjusted for major hospital characteristics, including Medicare Relative Risk score, patient volume, resident slots, intensive care unit level, and number of advanced specialty clinical programs (*2*). Proportions were modeled similarly but with a binomial distribution. All percentage changes are based on modeled rates. Statistical analyses were performed using Stata statistical software (release 15; StataCorp, LLC). This study was performed with approval from the University of Utah Institutional Review Board and the VA Salt Lake City Health Care System Research and Development Office.

## Results

**FIGURE 1:**
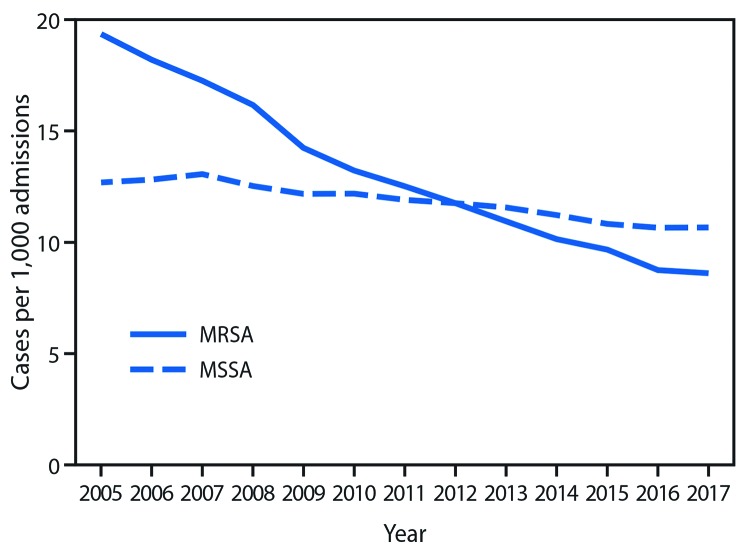
Rate* of *Staphylococcus aureus* infections among hospitalized patients, by methicillin resistance status — 130 Veterans Affairs medical centers, United States, 2005–2017 **Abbreviations:** MRSA = methicillin-resistant *Staphylococcus aureus*; MSSA = methicillin-susceptible *Staphylococcus aureus.* * Unadjusted.

**FIGURE 2:**
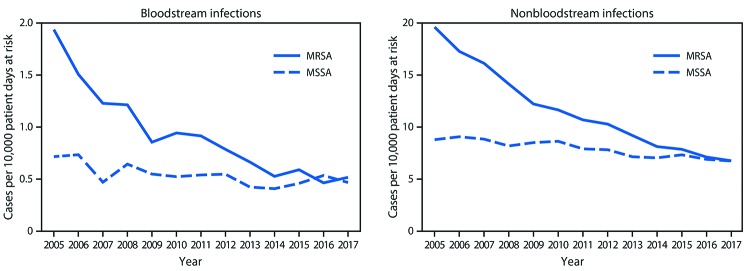
Hospital-onset *Staphylococcus aureus* bloodstream and nonbloodstream infection rates,* by methicillin resistance status — 130 Veterans Affairs medical centers, United States, 2005–2017 **Abbreviations:** MRSA = methicillin-resistant *Staphylococcus aureus*; MSSA = methicillin-susceptible *Staphylococcus aureus.* * Unadjusted.

The analysis included 130 VA hospitals. The overall rate of *S. aureus* infections decreased by 43% during 2005–2017 (4.7% annually, p<0.001) (Table). The reductions were driven primarily by decreases in MRSA infections, which declined by 55% (7.3% annual rate of decrease, p<0.001); MSSA infection rates decreased much more slowly, by 12% (1.2% annually, p = 0.003) (Figure 1). Hospital-onset MRSA infections decreased by 66% (p<0.001), and hospital-onset MSSA infections decreased 19% (p = 0.02); similar reductions were observed in both bloodstream and nonbloodstream infections (Figure 2).

**TABLE:** Changes in incidence of *Staphylococcus aureus* infections among hospitalized patients — 130 Veterans Affairs medical centers, United States, 2005–2017*

Infection characteristic	Overall change (%)	Average annual change (%)	p-value for trend
**All *S. aureus* infections**			
**Total (MRSA and MSSA)**	**-42.5**	**-4.7**	**<0.001**
Total MRSA	**-**54.6	**-**7.3	<0.001
Total MSSA	**-**12.2	**-**1.2	0.003
**Hospital-onset *S. aureus* infections**			
**All hospital-onset**	**-**70.2	**-**10.1	<0.001
MRSA	**-**65.7	**-**8.9	<0.001
MSSA	**-**18.7	**-**1.7	0.017
**Bloodstream**			
MRSA	**-**75.7	**-**11.8	<0.001
MSSA	**-**23.4	**-**2.2	0.357
**Nonbloodstream**			
MRSA	**-**64.1	**-**8.5	<0.001
MSSA	**-**18.8	**-**1.7	0.012
**Community-onset *S. aureus* infections**			
**All community-onset**	**-**27.5	**-**2.7	<0.001
MRSA	**-**40.6	**-**4.8	<0.001
MSSA	**-**0.4	**-**0.04	0.930
**Bloodstream**			
MRSA			
≤30 days postdischarge	**-**33.8	**-**3.8	0.022
31–365 days postdischarge	**-**15.2	**-**1.5	0.344
No discharge in last year	**-**11.1	**-**1.1	0.518
MSSA			
≤30 days postdischarge	**-**28.9	**-**3.1	0.139
31–365 days postdischarge	6.5	0.6	0.800
No discharge in last year	19.7	1.6	0.420
**Nonbloodstream**			
MRSA			
≤30 days postdischarge	**-**54.9	**-**7.2	<0.001
31–365 days postdischarge	**-**38.0	**-**4.4	<0.001
No discharge in last year	**-**36.5	**-**4.1	<0.001
MSSA			
≤30 days postdischarge	**-**0.1	**-**0.01	0.983
31–365 days postdischarge	10.5	0.9	0.159
No discharge in last year	0.6	0.06	0.916

**Abbreviations:** MRSA = methicillin-resistant *Staphylococcus aureus*; MSSA = methicillin-susceptible *Staphylococcus aureus*

* Based on multivariate analysis.

**FIGURE 3:**
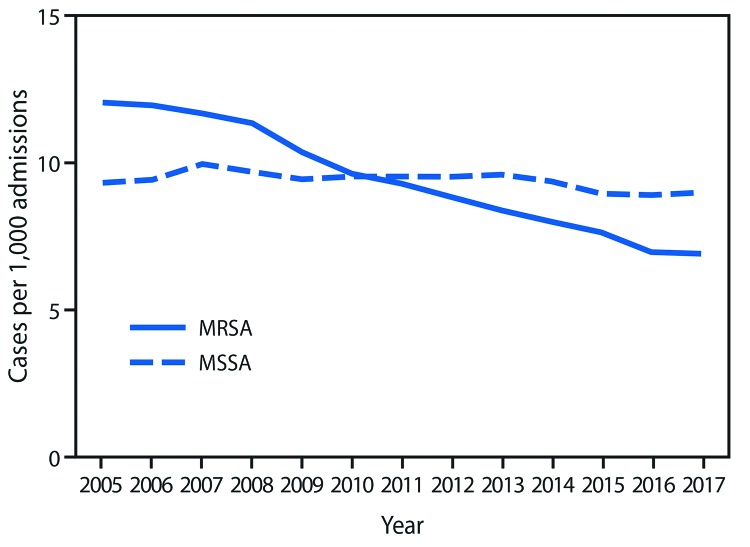
Community-onset *Staphylococcus aureus* infection rates,* by methicillin resistance status — 130 Veterans Affairs medical centers, United States, 2005–2017 **Abbreviations:** MRSA = methicillin-resistant *Staphylococcus aureus*; MSSA = methicillin-susceptible *Staphylococcus aureus.* * Unadjusted.

Among community-onset infections, overall MRSA infection rates decreased by 41% (p<0.001), and community-onset MSSA infection rates declined by 0.4% (p = 0.93) (Table) (Figure 3). The decreases in community-onset MRSA bloodstream and nonbloodstream infections were greatest among infections occurring within 30 days of hospital discharge (Table). Decreases in community-onset infections played a substantial role in overall *S. aureus* trends: reduction in community-onset MRSA infections accounted for 48% of the overall MRSA rate decreases, and 40% of decreases in overall *S. aureus* infection rates.

The rate of hospital-acquired MRSA colonization decreased 78% during the study period 6.8 per 1,000 patient-days at risk (2008) to 1.5 per 1,000 patient-days at risk (2017) (16.7% annually, p<0.001). When hospital-onset MRSA infection rates were stratified according to results of admission nasal surveillance tests, MRSA infection rates among patients whose admission screening tests were negative declined by 58% (9.7% annually, p<0.001). In contrast, the reduction among patients with positive admission screening tests was significantly less; MRSA infections decreased 31% (4.2% annually, p<0.001) (p<0.05 compared with patients with a negative admission test). Fluoroquinolone use did not change significantly between 2005 and 2008, but between 2009 and 2017, fluoroquinolone use rates decreased by 44% (annual decrease = 4.8% p<0.001).

## Conclusions and Comment

During 2005–2017, following introduction of a system-wide, multifaceted infection control intervention that included admission screening for nasal MRSA carriage and use of contact precautions for MRSA-colonized patients, VAMCs across the United States experienced a sharp decline in *S. aureus* infections among hospitalized patients. Most of the reductions were explained by decreases in MRSA; reductions in MSSA rates were more modest. Although the precise relationship between the observed trends and infection control interventions are difficult to demonstrate and likely complex, a careful examination of the potential mechanisms that could explain discordant MRSA and MSSA trends provides important insights for *S. aureus* prevention strategies.

One potential explanation for the discordant MSSA and MRSA trends is that the observed trends represent an artifact of differential detection bias, by which MRSA-infected patients would be progressively less likely than would MSSA-infected patients to have cultures obtained over the course of the study period. There is no obvious reason that likelihood of obtaining a diagnostic culture in patients with suspected infection would differ according to a provider’s clinical suspicion of MSSA versus MRSA, and there was no change in rate of diagnostic cultures obtained over the study period, nor was there any difference in diagnostic culture rate based on admission MRSA carriage status.

A second potential explanation is that shifts in *S. aureus* epidemiology might have influenced the observed trends. It has been suggested that downward temporal trends in community-associated infections caused by community-associated MRSA strains (e.g., USA300) might explain decreases in health care–associated MRSA. (*5*). Although strain data were not available for this analysis, data describing the national MRSA experience do not support this hypothesis. Population-based surveillance data from CDC’s Emerging Infections Program show that although rates of health care–associated MRSA infection rates have been declining, community-associated MRSA rates have remained unchanged since 2005 (*6*). In addition, almost all MRSA reductions resulted from decreases in USA100, a strain associated with health care system transmission (*7*). Conversely, only modest reductions were observed in USA300, a strain associated with community transmission. In the absence of replacement by other strains, this suggests that successful interruption of MRSA transmission in health care settings is an important contributor to national trends.

Infection control interventions might produce differential trends in MRSA and MSSA infection rates. Two broad approaches to preventing health care–associated infection include reducing the likelihood of invasive disease given colonization or exposure and decreasing transmission of pathogens (preventing infection by avoiding colonization or exposure in the first place). The VA system adopted both of these strategies. Similar to programs elsewhere, the VA system implemented bundled interventions designed to prevent device- and procedure-related infections (e.g., central line–associated bloodstream and surgical site infections). However, if such interventions were primarily responsible for the observed *S. aureus* trends, MSSA and MRSA rates would have been expected to have been affected approximately equally.

Other evidence also suggests decreased MRSA transmission as the primary mechanism for *S. aureus* reductions in VA hospitals. First, the discordance between MRSA and MSSA trends is consistent with mathematical modeling studies of health care transmission. Models predict that a decrease in overall transmission of bacterial pathogens in health care settings will result in disproportionately greater impact on strains having characteristics that provide a selective advantage for health care transmission, such as resistance to multiple antibiotics, including MRSA (*8*,*9*). Thus, the VA trends are consistent with decreased *S. aureus* transmission as the causative mechanism, regardless of whether improvements in infection control practices specifically targeted MRSA. Second, the rate of MRSA acquisition, a direct measure of MRSA transmission, decreased markedly during the course of the study. Third, reductions in hospital-onset MRSA infection were significantly greater among patients who were not carrying MRSA at the time of admission, suggesting that practices preventing acquisition of MRSA colonization had a greater impact than practices preventing progressing to infection among colonized patients. These findings are not consistent with the hypothesis that device- and procedure-associated prevention bundles, which are designed to prevent progression from colonization to infection, were primary drivers of *S. aureus* reduction in VA hospitals. Finally, the striking reductions in MRSA infection rates in the early postdischarge period are consistent with decreased acquisition during inpatient stays.

The mechanisms by which transmission was prevented are difficult to determine with precision, in part because multiple interventions were occurring simultaneously. It is highly plausible that the aggressive and targeted approach to preventing MRSA transmission (i.e., screening for MRSA carriage and implementation of contact precautions for all carriers) contributed to the pronounced decrease in MRSA infections. However, the discordant MRSA/MSSA trends might also be explained by infection control practices that prevent transmission of all bacterial pathogens, but do not specifically target MRSA, such as hand hygiene. A sustained decline in gram-negative rod bloodstream infections in the VA system after implementation of the MRSA prevention program was also observed (*10*). However, it is likely that contact precautions for MRSA-colonized patients contributed to this trend: another VA study showed that 31% of patients with multidrug-resistant gram-negative bacteria would have been under contact precautions because of a positive MRSA screen (*11*). Changes in antibiotic use could have contributed as well. There is evidence that fluoroquinolone use is associated with increased MRSA colonization (*12*), and the reduction in fluoroquinolone use could contribute to selective reduction in MRSA because it is more commonly fluoroquinolone-resistant than is MSSA. The VA did observe a substantial reduction in fluoroquinolone use, but the fluoroquinolone reductions did not begin until 2009, after substantial MRSA reductions had already occurred.

The findings in this report are subject to at least five limitations. First, the patient population in VAMCs is predominately male, although it is not clear that this characteristic would affect these findings. Second, the models used in this analysis did not include data regarding adherence to infection control practices; including such data might have provided additional insight into which components of the intervention might have had the most impact. Third, information about MSSA colonization was lacking, making it difficult to characterize MSSA transmission dynamics. Fourth, no information on MRSA or MSSA strain characteristics was available. Finally, simple exponential trends improve interpretability but might not always closely reflect trends in complex systems.

The significant reduction in *S. aureus* infection observed across VAMCs, driven primarily by a decrease in MRSA infection rates, offers important insights that can inform national *S. aureus* prevention strategy. Although the causal relationship between specific components of the VA-wide infection control intervention and the reduction in infection rates is difficult to determine with precision, it seems likely that decreased MRSA transmission played a substantial role. These data suggest that recent calls to withdraw infection control interventions (*5*) designed to prevent MRSA transmission, such as use of contact precautions, might be premature and inadvisable, at least until more is known about effective control of bacterial pathogen transmission in health care settings. Adherence to CDC recommendations (*13*) for antimicrobial stewardship, preventing device- and procedure-associated infections and interrupting transmission of health care–prevalent strains (e.g., use of contact precautions for MRSA) continue to be a mainstay of *S. aureus* prevention.

**Summary**

**What is already known about this topic?**

*Staphylococcus aureus* is an important cause of health care–associated infections and accounts for significant morbidity and mortality.

**What is added by this report?**

During 2005–2017, U.S. Department of Veterans Affairs medical centers across the United States experienced a sharp decline in *S. aureus* infections following introduction of a multifaceted infection control intervention. Most reductions were explained by decreases in methicillin-resistant *S. aureus* (MRSA). Decreased MRSA transmission likely played a substantial role.

**What are the implications for public health practice?**

These findings offer important insights informing *S. aureus* prevention strategy. Effective prevention strategies require a multifaceted approach, including efforts to prevent transmission of MRSA as well as efforts directed at infection prevention.
